# Case Report: Management of Cholestasis Associated With Congenital Syphilis

**DOI:** 10.3389/fped.2020.607506

**Published:** 2020-12-11

**Authors:** Kotaro Ogawa, Koya Kawase, Tokio Sugiura, Toshihiro Yasui, Seiya Yamagata, Tamao Watanabe, Yoshikazu Kawabe

**Affiliations:** ^1^Department of Pediatrics, Gamagori City Hospital, Gamagori, Japan; ^2^Department of Pediatrics and Neonatology, Nagoya City University Graduate School of Medical Sciences, Nagoya, Japan; ^3^Sugiura Kids Clinic, Hekinan, Japan; ^4^Department of Pediatric Surgery, Fujita Health University School of Medicine, Toyoake, Japan

**Keywords:** syphilis, congenital syphilis, *Treponema pallidum*, cholestasis, liver fibrosis

## Abstract

Cholestasis is a rare but life-threatening complication of congenital syphilis. However, standard management methods for this disease have not been established. Here, we report a case of congenital syphilis presenting with progressively worsening cholestasis, and we review the clinical features and management practices. In these cases, differentiation from other diseases presenting with cholestasis during the neonatal period, such as biliary atresia, is critical. In this regard, operative cholangiogram and histopathological analysis of the liver are required. Moreover, comprehensive genetic analysis can be useful. Although there is no specific treatment for cholestasis associated with congenital syphilis, appropriate nutritional management and supplementation with fat-soluble vitamins, especially vitamin K, should be provided. The severity of liver fibrosis may affect the prognosis of cholestasis associated with congenital syphilis. Therefore, attention should be paid to liver fibrosis in these patients.

## Introduction

Congenital syphilis (CS) occurs due to the transmission of *Treponema pallidum* from an infected mother to the fetus during pregnancy. Recently, an increasing number of patients with CS have been reported in many countries, resulting in serious global health concerns (1–3). CS causes a variety of complications, such as hemolytic anemia, meningitis, and osteochondritis. Although liver involvement is common in patients with CS, cases with cholestasis are limited. Cholestasis is a life-threatening complication of CS; however, standard management methods have not been established for this disease. Here, we report a case of CS presenting with progressively worsening cholestasis, and we review the clinical features and management practices.

## Case Description

A Japanese boy weighing 1,968 g was born *via* vaginal delivery. His mother was a 29-year-old primigravida woman. She had no prenatal care, including testing for syphilitic infection. The Apgar scores at 1 and 5 min were 5 and 7, respectively. The gestational age was estimated to be 36 weeks and 2 days using the Dubowitz method.

After birth, maternal serological tests for syphilis, rapid plasma reagin (RPR), and *T. pallidum* antibody (TPAb) test were positive. Maternal serological tests for human immunodeficiency virus (HIV), hepatitis B virus, and hepatitis C virus were negative. The infant had peeling skin on his face, hands, and feet. Hepatosplenomegaly and cervical lymphadenopathy were also observed. Initial laboratory examination showed a white blood cell count of 13.3 × 10^9^/L, a hemoglobin level of 12.8 g/dl, and a platelet count of 124 × 10^9^/L. C-reactive protein (CRP) and immunoglobulin M (IgM) levels were elevated to 4.24 and 331 mg/dl, respectively. The hepatic function test results were normal, with an aspartate aminotransferase (AST) level of 41 IU/L, alanine aminotransferase level of 4 IU/L, total bilirubin (TB) level of 2.9 mg/dl, and a direct bilirubin (DB) level of 1.1 mg/dl. The results of the coagulation studies were normal. Cytomegalovirus, rubella virus, herpes simplex virus, hepatitis B virus, hepatitis C virus, and HIV serological tests were negative. His RPR, TPAb, and fluorescent treponemal antibody-absorption serum tests were positive. Although cerebrospinal fluid (CSF) examinations showed normal lymphocytosis and protein levels, the CSF Venereal Disease Research Laboratory test was also reactive. Furthermore, histopathological examination of the placenta and umbilical cord revealed severe chorioamnionitis and necrotizing funisitis. Numerous *T. pallidum* cells were detected in Wharton jelly by immunohistochemistry, using an antibody against *T. pallidum*. Given these findings, he was diagnosed with CS and treated with penicillin G for 2 weeks.

**Figure 1:**
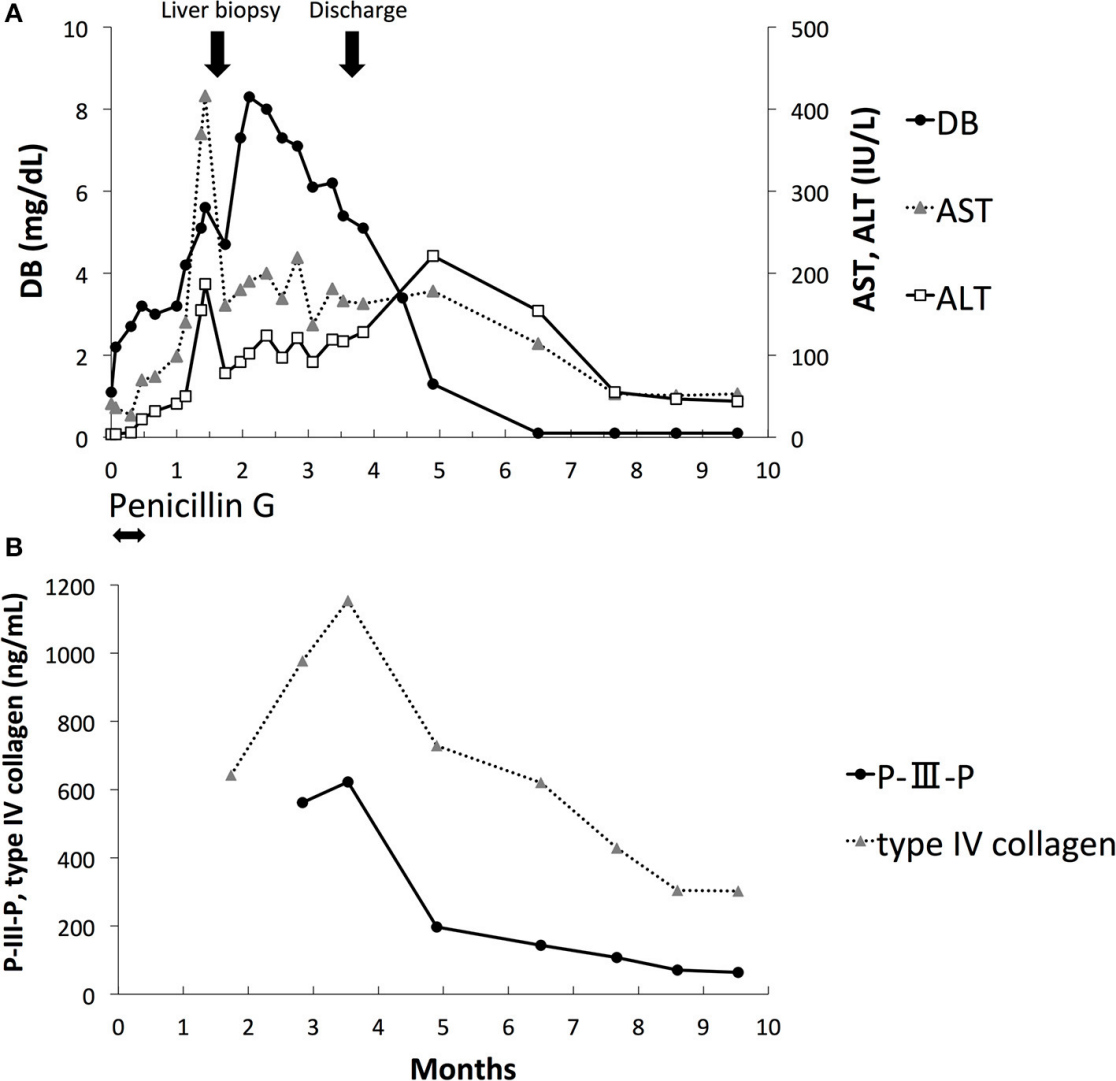
Laboratory data. **(A)** Direct bilirubin (DB), aspartate aminotransferase (AST), and alanine aminotransferase (ALT). **(B)** Procollagen type III peptide (P-III-P) and type IV collagen.

**Figure 2:**
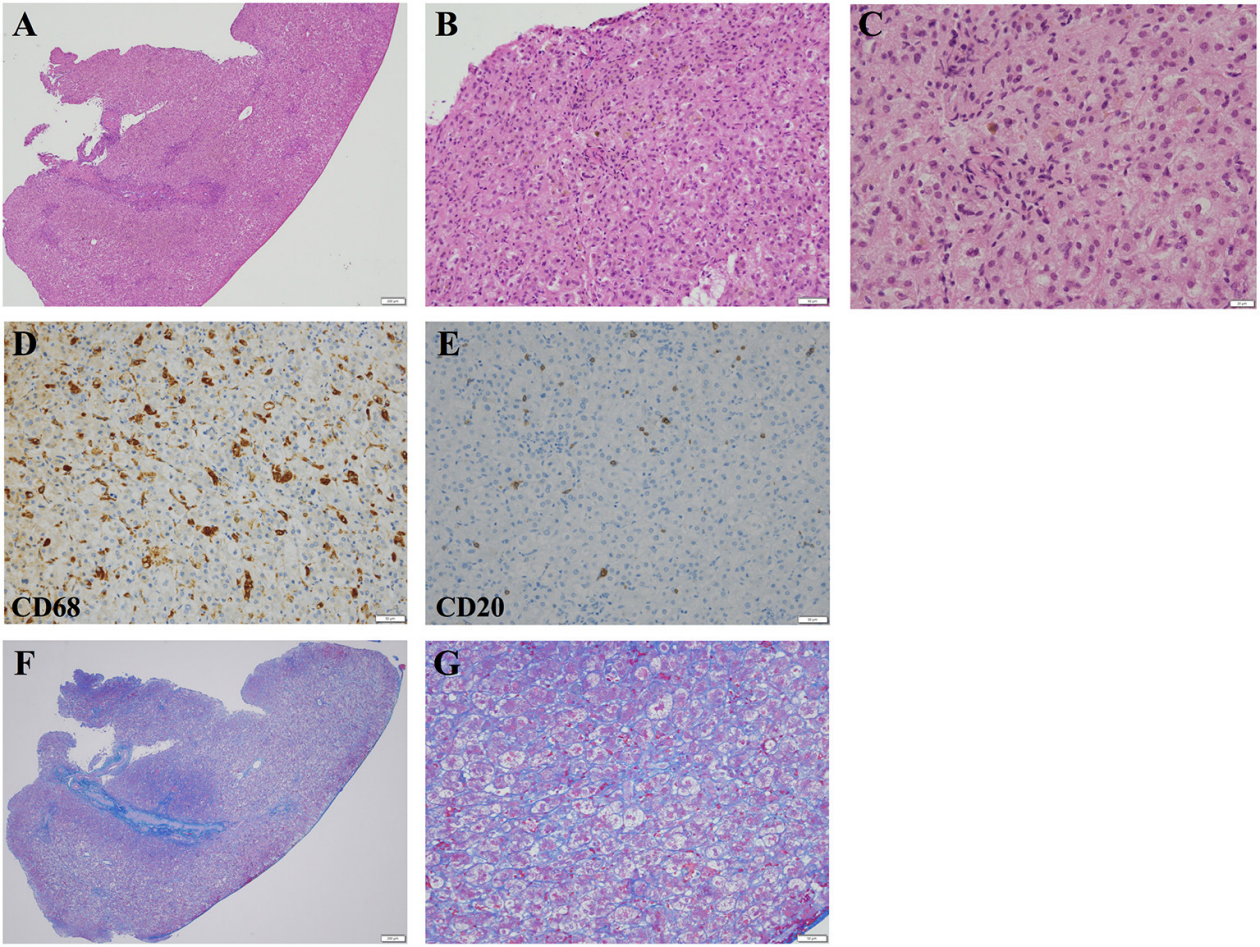
Light microscopic images of the liver biopsy specimen obtained at 1.5 months of age. **(A,B)** Hematoxylin and eosin staining demonstrated inflammatory cell infiltration and moderate intrahepatic cholestasis. **(A)** The scale bar represents 200 μm. **(B)** The scale bar represents 50 μm. **(C)** Higher-magnification images of hematoxylin and eosin-stained liver specimens demonstrating intrahepatic cholestasis. The scale bar represents 20 μm. **(D)** Most of the infiltrating inflammatory cells were CD68-positive macrophages. The scale bar represents 50 μm. **(E)** Small numbers of CD20-positive B lymphocytes were also observed. The scale bar represents 50 μm. **(F,G)** Azan staining demonstrated mild pericellular fibrosis. **(F)** The scale bar represents 200 μm. **(G)** The scale bar represents 50 μm.

After penicillin G therapy, CRP levels decreased rapidly to 0.09 mg/dl at day 14. However, he developed cholestasis at 1 month of age, with elevated serum TB (7.0 mg/dl), DB (5.6 mg/dl), AST (416 IU/L), ALT (187 IU/L), γ-glutamyl transferase (139 IU/L, reference range: 13 to 64 IU/L), and total serum bile acid (141.8 μmol/L, reference range: <10 μmol/L) levels. Common causes of neonatal cholestasis, such as hypothyroidism, galactosemia, and α1-antitrypsin deficiency, were excluded by blood examination. He needed to receive a formula containing medium-chain triglycerides to achieve adequate body weight gain. He was treated with ursodeoxycholic acid, and supplementation with vitamins D and K was started. However, his cholestasis progressively worsened (Figure 1A), and acholic stools were observed. His gallbladder was found to be normal and no triangular cord sign was observed by ultrasonography, suggesting a lower possibility of biliary atresia (BA). However, considering that ultrasonography cannot completely rule out BA, surgical intervention was performed at 1.5 months of age. Operative cholangiography showed a normal gallbladder and biliary tree, thus excluding BA. Liver pathology tests showed intrahepatic cholestasis with a few bile plugs within the bile ducts (Figures 2A–C). Moderate inflammatory cell infiltration was also observed, mainly consisting of CD68-positive macrophages and small numbers of CD20-positive B lymphocytes (Figures 2D,E). Azan staining showed mild pericellular fibrosis surrounding hepatocytes, with slight fibrosis in the portal vein area (Figures 2F,G). Bile duct paucity was not observed, and *T. pallidum* was not detected in the liver. To further delineate the etiology, we also performed targeted next-generation DNA sequencing for neonatal and infantile cholestasis (4), and no significant causative mutations were identified. Therefore, cholestasis was determined to be associated with CS.

DB, AST, and ALT levels peaked at 2 months of age and improved gradually thereafter (Figure 1A). The serum RPR test results were negative at 2 months of age. The levels of serum markers of liver fibrosis, including procollagen type III peptide (P-III-P, 622 ng/mL, reference range: 3.6 to 9.5 ng/mL) and type IV collagen (1,154 ng/mL, reference range: <150 ng/mL), increased at 3 months of age; however, they gradually decreased thereafter (Figure 1B). He was discharged at 3.5 months of age with DB, ALT, and AST levels of 5.4 mg/dl, 117 IU/L, and 166 IU/L, respectively. The level of DB was within the normal range at 5 months of age, and the levels of AST and ALT were within the normal range at 9 months of age. At the 10-month follow-up, he had no apparent sequelae of CS.

Our case highlights the importance of correct differential diagnosis and management of cholestasis associated with CS. However, the long-term prognosis and effects of cholestasis associated with CS on liver function are still unknown.

## Discussion

Cholestasis is a rare but life-threatening complication of CS. In typical cases, the levels of DB and liver enzymes progressively increase after the initiation of penicillin G treatment and persist for several weeks (5–7). Liver pathology demonstrates inflammatory cell invasion, giant cell transformation, hepatocellular cholestasis, and fibrosis (8). Usually, *T. pallidum* is not detected in the liver. The pathogenesis of cholestasis is not currently known; however, toxic reactions to the products of treponemal lysis have been speculated to be the cause (5, 9). Although cholestasis naturally improves in most cases, several fatal cases, due to intracranial or gastrointestinal hemorrhage with severe liver fibrosis, have been reported (10, 11).

Although penicillin G is established as an effective treatment for CS, there is no consensus on the management of cholestasis associated with CS. In cases that progressively worsened, such as in our case, the exclusion of other diseases presenting with cholestasis in the neonatal period becomes critical. In particular, BA should be excluded as early as possible using an operative cholangiogram. Liver biopsy should be performed to exclude several diseases with specific pathological findings of the liver, such as the paucity of bile ducts. Comprehensive genetic analysis based on next-generation DNA sequencing (4) can be useful for distinguishing between genetic diseases, such as Alagille syndrome, progressive familial intrahepatic cholestasis, Dubin–Johnson syndrome, and neonatal intrahepatic cholestasis caused by citrin deficiency.

There is currently no specific treatment for cholestasis associated with CS. Therefore, it is of utmost importance to provide adequate supportive therapy for these patients. Growth failure can occur, as seen in our case, secondary to impaired absorption of fats, impaired metabolism of proteins and carbohydrates, and increased metabolic demand. Feeding milk containing medium-chain triglycerides is useful because biliary hyposecretion makes it difficult to digest normal triglycerides. The absorption of fat-soluble vitamins is also impaired. In particular, vitamin K should be supplied to prevent fatal hemorrhage. In addition, coagulation function should be monitored repeatedly.

The severity of liver fibrosis may affect the prognosis of cholestasis associated with CS. P-III-P and type IV collagen are known liver fibrosis markers, and it has been reported that the levels of these markers are significantly elevated in fatal cases of transient myeloproliferative disorder of Down syndrome with severe liver fibrosis (12, 13). In our case, the liver pathology at 1.5 months of age showed mild fibrosis. The levels of serum P-III-P and type IV collagen were elevated; however, they decreased gradually after 3 months of age. Although the concordance of the levels of these liver fibrosis markers and liver histology is still unknown, our case suggests the utility of repeated evaluation of these markers for the noninvasive monitoring of liver fibrosis and the prediction of prognosis. Transient elastography (TE) has recently attracted attention as a new tool for the noninvasive assessment of liver fibrosis (14, 15). Wu et al. reported that TE values were correlated with METAVIR liver histology scores in subjects with BA (16). TE could be useful for the noninvasive assessment and follow-up of liver fibrosis in patients with CS-associated cholestasis. However, the correlation between TE values and liver histology in cholestasis associated with CS is still unknown.

Given that the number of patients with CS is increasing in many countries, the management of cholestasis associated with CS is becoming more important. Further research is needed to establish methods for the early identification of fatal cases and effective treatments for these patients. In addition, case accumulation is needed to elucidate the long-term prognosis and the effects of cholestasis associated with CS on liver function in these patients.

## Data Availability Statement

The original contributions generated for this study are included in the article/supplementary materials, further inquiries can be directed to the corresponding author/s.

## Ethics Statement

The studies involving human participants were reviewed and approved by the Ethics Committee of Gamagori City Hospital. Written informed consent to participate in this study was provided by the patient's legal guardian or next to kin.

## Consent for Publication

Informed written consent was obtained from the patient's legal guardian or next of kin for publication of this case report (including all data and images).

## Author Contributions

KO, KK, TS, SY, TW, and YK managed the patient and contributed to the conception of the study. TY performed the surgical procedure. KO, KK, and TS drafted the manuscript. All authors have read and approved the final manuscript.

## Conflict of Interest

The authors declare that the research was conducted in the absence of any commercial or financial relationships that could be construed as a potential conflict of interest.

## Abbreviations

CS: congenital syphilis
RPR: rapid plasma reagin
TPAb: *Treponema pallidum* antibody
HIV: human immunodeficiency virus
CRP: C-reactive protein
IgM: immunoglobulin M
AST: aspartate aminotransferase
ALT: alanine aminotransferase
TB: total bilirubin
DB: direct bilirubin
CSF: cerebrospinal fluid
BA: biliary atresia
P-III-P: procollagen type III peptide
TE: transient elastography.
